# The Usage of Cryopreserved Reproductive Material in Cancer Patients Undergoing Fertility Preservation Procedures

**DOI:** 10.3390/cancers15225348

**Published:** 2023-11-09

**Authors:** Katarzyna Wnuk, Jakub Świtalski, Wojciech Miazga, Tomasz Tatara, Urszula Religioni, Paweł Olszewski, Anna Augustynowicz

**Affiliations:** 1 Department of Health Policy Programs, Department of Health Technology Assessment, Agency for Health Technology Assessment and Tariff System, 00032 Warsaw, Poland; 2 School of Public Health, Centre of Postgraduate Medical Education of Warsaw, Kleczewska 61/63, 01826 Warsaw, Poland; 3 Department of Health Economics and Medical Law, Faculty of Health Sciences, Medical University of Warsaw, 01445 Warsaw, Poland; jakub.switalski@wum.edu.pl; 4 Department of Public Health, Faculty of Health Sciences, Medical University of Warsaw, 02091 Warsaw, Poland; 5 Medical Faculty, Lazarski University, 02662 Warsaw, Poland

**Keywords:** reproductive health, oncofertility, fertility preservation, cryopreservation, cancer

## Abstract

**Simple Summary:**

Cancer treatment, in particular with gonadotoxic potential, may affect the fertility of cancer patients and cause temporary or permanent damage to the reproductive organs and glands that control fertility. Taking into account that some patients ultimately do not lose fertility during treatment, some do not survive cancer therapy, and some do not decide to use cryopreserved reproductive material, the review analysed the percentage of usage of cryopreserved reproductive material collected before treatment to preserve the fertility of patients after cancer treatment. The obtained review results indicate a low return/usage rate of cryopreserved reproductive material among both women and men. This review highlights potential organizational issues related to storage costs, space needed, and the use or disposal of stored material. Considering the increase in the number of cancer patients, the scale of this problem may turn out to be significant in the coming years.

**Abstract:**

Background: Many cancer treatment methods can affect fertility by damaging the reproductive organs and glands that control fertility. Changes can be temporary or permanent. In order to preserve the fertility of cancer patients and protect the genital organs against gonadotoxicity, methods of fertility preservation are increasingly used. Considering that some patients ultimately decide not to use cryopreserved reproductive material, this review analysed the percentage of post-cancer patients using cryopreserved reproductive material, collected before treatment as part of fertility preservation. Methods: A systematic search of studies was carried out in accordance with the Cochrane Collaboration guidelines, based on a previously prepared research protocol. The search was conducted in Medline (via PubMed), Embase (via OVID), and the Cochrane Library. In addition, a manual search was performed for recommendations/clinical practice guidelines regarding fertility preservation in cancer patients. Results: Twenty-six studies met the inclusion criteria. The studies included in the review discussed the results of cryopreservation of oocytes, embryos, ovarian tissue, and semen. In 10 studies, the usage rate of cryopreserved semen ranged from 2.6% to 21.5%. In the case of cryopreserved female reproductive material, the return/usage rate ranged from 3.1% to 8.7% for oocytes, approx. 9% to 22.4% for embryos, and 6.9% to 30.3% for ovarian tissue. In studies analysing patients’ decisions about unused reproductive material, continuation of material storage was most often indicated. Recovering fertility or death of the patient were the main reasons for rejecting cryopreserved semen in the case of men. Conclusion: Fertility preservation before gonadotoxic treatment is widely recommended and increasingly used in cancer patients. The usage rate is an important indicator for monitoring the efficacy of these methods. In all of the methods described in the literature, this indicator did not exceed 31%. It is necessary to create legal and organizational solutions regulating material collection and storage and to create clear paths for its usage in the future, including by other recipients.

## 1. Introduction

According to the definition by the International Classification of Diseases 11th Revision, female infertility (ICD-11: GA31) is a “disease of the reproductive system defined by the failure to achieve a clinical pregnancy after 12 months or more of regular unprotected sexual intercourse” [1]. Male infertility (ICD-11: GB04) is defined as “any disorder of the reproductive system affecting males, characterized by dysfunctionalities in the ejection of semen or an abnormal absence in the measurable level of sperm in semen” [2].

According to the data of the International Agency for Research on Cancer (based on the GLOBOCAN estimates), in 2020 there were approximately 3.1 million new cases of cancer among people of reproductive age (aged 15–49) [3]. Many cancer treatment methods can affect fertility by damaging the reproductive organs and glands that control fertility. Changes can be temporary or permanent. For some patients, infertility can be one of the most difficult and distressing long-term effects of cancer treatment. It is important to assess how the planned therapy may affect fertility before starting treatment [4,5,6,7,8].

In 2006, Dr. Teresa K. Woodruff of Northwestern University launched a new field of medicine that combines oncology with reproductive health, calling it “oncofertility”. The interdisciplinary initiative “Oncofertility Consortium”, established by her, focuses on problems related to healthcare and the quality of life of young cancer patients, in particular on issues related to fertility after cancer [9].

In order to preserve the fertility of cancer patients and protect the genitals against gonadotoxic effects (chemotherapy and/or radiotherapy), the following are used in women: oocyte cryopreservation, embryo cryopreservation, ovarian tissue cryopreservation, gonadal shielding, ovarian transposition (oophoropexy), surgical techniques (trachelectomy), and treatment with gonadotropin-releasing hormone agonist (GnRHa) [10,11,12,13,14,15]. In men, fertility preservation techniques may include semen cryopreservation, gonadal shielding, testicular sperm extraction, partial ovariectomy, and testicular tissue cryopreservation [16,17,18]. For the purposes of our article, reproductive material is defined as human gametes, embryos, and tissues.

Factors affecting the patient’s decision regarding the usage of the above-mentioned methods may include personal beliefs, religious and cultural limitations, prognosis, the patient’s knowledge of fertility preservation methods, as well as the method of disseminating information about the possibility of fertility preservation by medical personnel [19,20,21,22,23].

Due to the increasing usage of fertility preservation methods and emerging discussions on the ethics of the above-mentioned procedures (in terms of embryo freezing) and subsequent usage of cryopreserved materials, a review of scientific evidence describing the reproductive results obtained as a result of using fertility preservation procedures among cancer patients was performed. Considering that some cancer patients do not lose fertility during treatment, some do not survive cancer therapy, and some ultimately do not decide to use cryopreserved reproductive material, this review analyses the percentage of patients using cryopreserved reproductive material collected before treatment to preserve fertility.

Bearing in mind the above, the main objective of the article is to analyse the usage of cryopreserved reproductive material collected from patients before cancer treatment as part of fertility preservation.

## 2. Materials and Methods

The analysis was carried out based on the results of available studies. The search was based on a detailed protocol developed prior to the commencement of this work. It takes into consideration the criteria for including studies in the review, the search strategy, the method of selecting studies, and the planned methodology for conducting the analysis. The review was performed according to the Cochrane Collaboration guidelines [24].

The analysis included clinical trials that met the criteria regarding:

- Population: people diagnosed with cancer;
- Interventions: fertility preservation (cryopreservation of oocytes, embryos, ovarian tissues, semen, and testicular tissues);
- Alternative technologies (comparators): not restricted;
- Outcome: return/usage rate, decisions/disposal of unused reproductive material;
- Types of studies: systematic reviews, experimental or observational studies.

The review did not include studies that analysed fertility preservation methods in patients undergoing cancer treatment that did not require the collection and storage of reproductive material, e.g., gonadal protection, hormone therapy, conserving treatment, and ovarian transposition.

The following sources of medical information were searched for studies published in the last 10 years (i.e., since 2013): Medline (via PubMed), Embase (via Ovid), and The Cochrane Library. The search of the databases was carried out on 12 June 2023 in accordance with the search strategies presented in the Supplementary Materials. In addition, publications with clearly defined endpoints (specified in the PICO scheme), included in the bibliography of studies found in the review, were also included in the analysis. In addition, manual searches of clinical practice recommendations/guidelines regarding fertility preservation in cancer patients and grey literature were performed (searches included TRIP Database and Google Scholar).

At all stages of the review, studies were selected by two analysts working independently (K.W. and J.Ś.). Inconsistencies were resolved by consensus, with the participation of a third independent analyst (W.M.).

The review included publications that clarified the presented methodology, were of high quality, and had a low risk of error.

Based on the included publications, data from each publication were summarized in terms of two main outcomes (return/usage rate, and decisions/disposal of unused reproductive material). In terms of the usage of cryopreserved reproductive material in cancer patients, the results were presented in tabular and descriptive form (separately for women and men). Reproductive material that was cryopreserved was analysed and the number of used materials/return for materials, number of patients who cryopreserved material, and the return/usage rate were indicated. In the case of the decisions/disposal of unused reproductive material outcome, information was presented regarding the number of patients who cryopreserved material, reproductive material, disposal of unused cryopreserved material, and, additionally, usage rate.

## 3. Results

**Figure 1:**
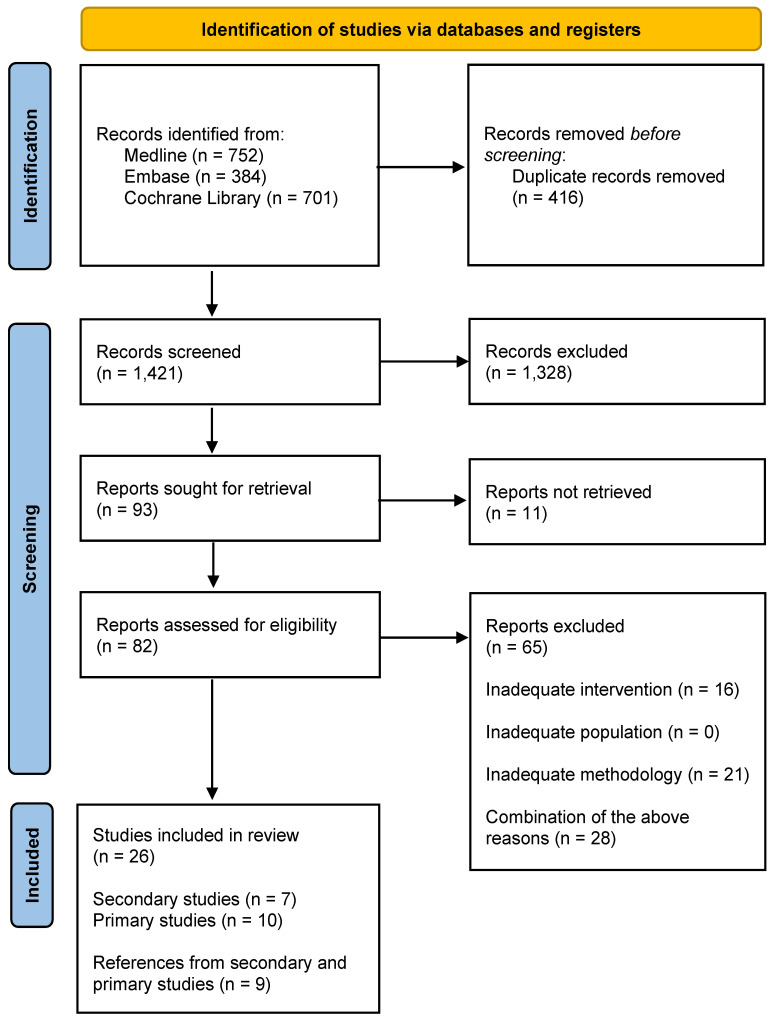
PRISMA flow diagram.

The study selection stages are presented in Figure 1. The list of publications included and excluded, along with the reasons for exclusions from the review, can be found in the Supplementary Materials Table S1.

The most common reasons for the exclusion of studies from the analysis were issues related to methodology (lack of proper description of the materials and methods, incorrect synthesis of review results, misinterpretation of statistical results) and intervention (preserving fertility among people without cancer).

### 3.1. The Usage of Cryopreserved Reproductive Material in Cancer Patients

#### 3.1.1. Women

As part of this review, sixteen publications were found indicating the percentage of female cancer patients who underwent fertility preservation procedures, who after their treatment decided to use cryopreserved reproductive material [25,26,27,28,29,30,31,32,33,34,35,36,37,38,39,40].

According to the results of the Fraison 2023 meta-analysis, 4.6% of women used cryopreserved oocytes for fertilization under assisted reproductive technology (ART), and approx. 9% of women decided to get pregnant using cryopreserved embryos [25]. In turn, the Xu 2023 meta-analysis, based on twenty-six observational studies, analysing the reproductive results of female cancer patients undergoing FP procedures, indicated a return rate of 7.9% in total for cryopreserved oocytes, embryos, and ovarian tissues [26]. On the other hand, in the Sheshpari 2019 systematic review assessing the efficacy of fertility preservation as part of the ovarian tissue collection and cryopreservation procedure, it was indicated that 30.3% of women returned to have the OTT procedure after the completion of cancer treatment [34].

As part of the studies analysed in the found reviews, the return rate was 3.1% to 7.5% for cryopreserved oocytes [32,35,38], 18.75% for cryopreserved embryos [27,37], and 12% for cryopreserved oocytes and embryos together [30,40].

In a 25-year prospective cohort study, 8.7% of women returned to attempt pregnancy using their stored oocytes [28]. Moreover, in other studies, the return rate for cryopreserved reproductive material ranged from 6.9% (for ovarian tissue) [33] to 22.4% (for cryopreserved embryos) [39].

Below are the individual results of the studies regarding the percentage of women who used reproductive material after completing cancer treatment as part of fertility preservation (Table 1).

#### 3.1.2. Men

As part of the analysis of publications found in the review, studies were also indicated that determined the percentage of men undergoing the FP procedure who, after completing cancer treatment, returned and used cryopreserved reproductive material (semen) for fertilization under ART (n = 10) [41,42,43,44,45,46,47,48,49,50].

According to the results of individual studies, the return/usage rate did not exceed 22%, and it ranged from 6.5% [49] to 21.5% [48]. However, in a meta-analysis of 30 observational studies covering a population of 11,798 men, the percentage of people who used cryopreserved semen was 8.5% [45].

Below are the individual results of the studies on the number of men undergoing cancer treatment who underwent cryopreservation as part of FP and the return/usage rate of the material under assisted reproduction after the completion of cancer treatment (Table 2).

#### 3.1.3. Decisions and Disposal of Unused Reproductive Material

On the basis of five studies, the results regarding the decision to transfer or reject unused cryopreserved reproductive material were presented [36,43,45,46,50].

Depending on the observation period, cancer patients most often continued to store the cryopreserved material (from 34.3% to 55.4% of the cryopreserved material) [36,46,50]. In turn, the Moravek 2018 retrospective cohort study indicated that female patients who stored unused cryopreserved oocytes or embryos decided to donate their reproductive material for research purposes or decided to donate oocytes and/or embryos to another couple or family members [36].

In turn, the main reasons for the rejection of the cryopreserved material (semen) were regaining fertility through the return of reproductive functions after cancer treatment [43,46] and the patient’s death [43,46,51].

The individual results of the studies concerning the decisions and disposal related to the unused reproductive material of cancer patients are presented below (Table 3).

In the Sankara-Narayana 2019, Muller 2016, and Johnson 2013 studies, the results concerning the disposal of unused cryopreserved material and the usage rate refer to all banked samples (100%). In the Ferrari 2016 study, the discard rate included a meta-analysis of eleven studies (n/N = 691/4291), and the usage rate refers to the number of all patients included in the meta-analysis (n/N = 974/11,798). In the case of the Moravek 2018 study, the results refer to the material disposal selected at the time of cryopreservation.

## 4. Discussion

Based on the results of studies found as part of the systematic search, the usage of cryopreserved reproductive material, collected from patients before cancer treatment as part of fertility preservation, was analysed. The publications included in the review covered various fertility preservation methods (including cryopreservation of eggs, embryos, ovarian tissue, and semen).

The usage rate varied depending on the study and the type of cryopreserved material. The usage rate of cryopreserved sperm ranged from 2.6% to 21.5% [41,42,43,44,45,46,47,48,49,50]. Considering cryopreserved reproductive material in women, some studies analysed the use of oocytes, embryos, and ovarian tissues together. Taking into account this type of study, the return/usage rate ranged from 7.9% to 21.2% [26,29,31,33]. Bearing in mind the studies that showed the results for individual types of material, it can be seen that the return/usage rate ranged from 3.1% to 8.7% in the case of oocytes [25,28,32,35,38], from approx. 9% to 22.4% in the case of embryos [25,27,37,39], and from 6.9% to 30.3% in the case of ovarian tissues [33,34].

Considering the above results, attention should be paid to potential organizational problems related to the costs of storage, the space needed, and the usage or disposal of material (which may be particularly problematic from an ethical point of view in the case of cryopreserved embryos) [52]. Considering the increase in the number of cancer patients, the scale of this problem may turn out to be significant in the coming years. The small number of national oncofertility registries is also problematic and translates into potential organizational problems and limited possibilities for using cryopreserved materials [51,52].

In two studies included in this review, the control groups were non-oncological women [28] and women who decided to undergo elective fertility preservation [35]. According to the results of the Porcu 2022 study, the return rate was lower in the group of women with cancer compared to the group of women who had not been diagnosed with cancer—8.7% vs. 83.5% (44/508 vs. 870/1042) [28]. In the second study, the return rate was statistically significantly higher in the elective fertility preservation group compared to the oncological women intervention group (12.1% vs. 7.4%; *p* < 0.05). Therefore, there is a visible difference in the return rate in the case of healthy women and women suffering from cancer. The above situation may be related primarily to the death of oncological patients and the lack of loss or recovery of the functions of reproductive organs after recovery from the disease. These situations reduce the level of use of frozen reproductive materials. In the case of women not suffering from cancer, it should be borne in mind that freezing reproductive materials is most often associated with diagnosed infertility and planned in vitro fertilization procedures. The use of frozen eggs/embryos will therefore be more frequent (including due to the sometimes necessary repetition of in vitro fertilization procedures). To complete the analysis, databases and websites of scientific societies were manually searched for clinical practice guidelines on fertility preservation against gonadotoxic therapy. Recommendations from the last 10 years were sought. The main conclusions are presented below.

The vast majority of recommendations indicate the need to inform all people diagnosed with cancer about the potentially harmful impact of the planned treatment on fertility. Before the commencement of treatment, the available fertility preservation methods should be discussed with the patient [53,54,55,56,57,58,59,60,61,62,63,64,65,66] and information and educational materials provided should be age-appropriate [53,59,60]. Patients interested in fertility preservation should be referred to fertility specialists [53,54,55,56,57,60,62,67]. The recommendations also emphasise that comprehensive care for cancer patients in terms of fertility preservation should include psychosocial support [53,59,62,64].

Almost all documents found indicate cryopreservation of oocytes and/or embryos as a method of fertility preservation in women of reproductive age, which should be implemented before commencing cancer treatment with gonadotoxic potential [53,54,55,56,57,59,60,61,62,63,64,65,66,67,68]. Most of the recommendations are consistent in that if the implementation of the above-mentioned methods is not possible (e.g., in pre-pubescent patients or when the commencement of cancer therapy should not be delayed), cryopreservation and subsequent auto-graft of ovarian tissue are recommended [53,54,55,56,57,60,61,62]. In some guidelines, this method was considered experimental [55,58,66,67] and the need to consider the individual risk associated with cancer reimplantation must be taken into account [53,60,63].

In the population of men and boys after puberty, in whom cancer treatment with gonadotoxic potential is planned, the basic method of preserving fertility is semen cryopreservation [53,54,55,56,57,58,60,61,62,63,64,65,66,67,68]. In a situation where they are unable to provide a semen sample themselves, testicular sperm extraction or electroejaculation is recommended [56,57,63].

Cryopreservation of testicular tissue is an option for pre-pubertal fertility preservation in boys. However, the societies emphasize that this is an invasive and experimental method, and should only be considered in clinical trials [53,54,55,56,57,61,62,63,66].

When analysing the issue of cryopreservation of reproductive materials, the costs associated with this procedure should also be taken into account. In one publication, the authors attempted to estimate the costs of fertility preservation activities among women undergoing cancer treatment. Cost estimates in this case referred to oocyte cryopreservation. The publication did not indicate the general cost-effectiveness threshold for the implementation of the discussed intervention; however, based on the collected data from the systematic search, it was estimated that the total cost of one cycle (including collection, cryopreservation, storage, and fertilization with implementation) ranges between USD 7000 and USD 14,000. In addition, pharmaceuticals used in the collection of oocytes from women also play an important role. In this case, the cost of drugs for one egg cell ranges from USD 2000 to USD 7000. An additional element significantly affecting such high costs of oocyte cryopreservation involves the costs associated with long-term storage of the collected cells. In this case, annual fees for stored cells range from USD 350 to USD 600 [58].

In another publication, the authors attempted to estimate the cost-effectiveness of semen cryopreservation as a method of preserving the fertility of men with testicular cancer. The publication did not indicate the general cost-effectiveness threshold for semen cryopreservation; however, according to estimates, the average cost of the procedure using a single vial was USD 754 in the first year of intervention. In turn, each subsequent year of storage of the collected reproductive material costs an average of USD 343 [69].

Taking into account the high level of non-use of frozen reproductive materials among people with cancer, attention should be paid to the validity of the distinction between gametes and embryos in legal acts, with particular emphasis on the handling of each of these reproductive materials. For example, in the Polish Act on the Treatment of Infertility, a reproductive cell is defined as “a human male reproductive cell (human sperm) or a human female reproductive cell (human egg cell) intended for use in a medically assisted procreation procedure”. In turn, an embryo is defined as “a group of cells resulting from the extracorporeal fusion of female and male reproductive cells, from the completion of the process of fusion of the germ cell nuclei (karyogamy) to the moment of implantation in the uterine mucosa” [70].

Pursuant to the provisions of the Act, reproductive cells may be destroyed or donated for research purposes at the express request of the donor at any time. In turn, embryos created from the collected reproductive cells are transferred for embryo donation (“transfer of an embryo for the purpose of using it in a medically assisted procreation procedure in a recipient who is not a donor of female reproductive cells and is not married or in cohabitation with a donor of male reproductive cells reproductive cells from which the embryo was created”) after the expiry of the deadline specified in the agreement concluded between the reproductive cell and embryo bank and the donors. However, this deadline cannot be longer than 20 years from the date on which the embryos were transferred to the bank of reproductive cells and embryos for their storage. Embryo donation may also take place in the event of the death of both embryo donors or—if the embryo was created as a result of non-partner donation—the death of the recipient and her husband or a person in cohabitation with her [70]. However, the problem in this case may be the increasing number of unused and stored embryos.

A very important issue is the conscious decision of people with cancer and their partners regarding the method of future fertility protection they choose. In potential legal cases described in the literature, there may be situations where the partner does not consent to the woman’s use of embryos after the couple breaks up [71,72]. For some women, egg freezing might be a better choice, which would avoid the situation described above.

Another aspect is the need to carry out the in vitro fertilization procedure for people who decide to freeze and later use eggs, embryos, or sperm. For some people, carrying out such a procedure may be unacceptable due to their beliefs. The solution for these people may be collecting and freezing ovarian tissue and its subsequent autotransplantation. In the case of men, the solution of freezing testicular tissue is not a procedure with proven effectiveness, therefore other procedures are performed in their case (mainly sperm freezing), which consequently lead to in vitro fertilization.

## 5. Conclusions

Fertility preservation in the case of gonadotoxic treatment is widely recommended and increasingly used in cancer patients. The basic methods include cryopreservation of oocytes and embryos in women and semen in men. The usage rate is an important indicator for monitoring the efficacy of these methods. In all of the methods described in the literature, this indicator did not exceed 31%.

Unused cryopreserved reproductive material was usually stored further. Some of the material was donated for scientific purposes or to other people in need. Important factors that prevent the usage of cryopreserved material include the patient’s death or regaining fertility through the return of reproductive functions after cancer treatment.

It is necessary to create legal and organizational solutions regulating the collection and storage of material and to create clear paths for its usage in the future, including by other recipients.

## 6. Review Limitations

Only publications in English were included in the review. The studies were characterised by high heterogeneity (e.g., different methods of presenting the analysed data or differences in the scope of the applied interventions). In the context of the analysed endpoints, individual studies covered a varied follow-up period, which ranged from several months to several decades (in the case of retrospective studies).

## Figures and Tables

**Table 1:** The usage of cryopreserved reproductive material by women after completing cancer treatment.

Author/Year (Type of Study)		Participant Age Range (Years) or Mean/Median Age	Reproductive Material	Number of Used Materials/Return for Materials	Number of Patients Who Cryopreserved Material	Return/ Usage Rate
Fraison 2023 (SR) [25]		15–45	Oocytes	178	3851	4.6%
		25.4–37.5	Embryos	160	1779	~9%
Xu 2023 (SR) [26]		18–35 (22 studies) >35 (1 study) <18 (1 study)	Oocytes, embryos, ovarian tissues	558	7037	7.9%
Ozcan 2022 (SR) [27] Dolmas 2015 (PR)# [37]		21–41 (mean age 30 ± 4.6)	Embryos	9	48	18.75%
Bonardi 2020 (SR) [30] Johnson 2013 (PR)# [40]		19–43 (mean age 31.2)	Oocytes and/or embryos	6	50	12%
Wang 2020 (SR) [32]	Cobo 2018 (PR)# [35]	mean age 32.3 ± 3.5	Oocytes	80	1073	7.5%
	Martinez 2014 (PR)# [38]	15–43 (mean age 31.9)		11	357	3.1%
Sheshpari 2019 (SR) [34]		mean age for transplantation 31	Ovarian tissue	210	693	30.3%
Porcu 2022 (PR) [28]		29.4 ± 4	Oocytes	44	508	8.7%
Marklund 2021 (PR) [29] Marklund 2020 (PR)# [31]		21–42	Oocytes, embryos, ovarian tissues	99 (usage or FP re-guidance)	468	21.2%
Rodriguez-Wallberg 2019 (PR) [33]		3–42 (mean age 27.4 ± 8.0)	Oocytes and/or embryos, ovarian tissues	71	563	12.6%
			Ovarian tissue	2	29	6.9%
Moravek 2018 (PR) [36]		15–42	Oocytes, embryos	21	204	10.3%
Courbiere 2013 (PR) [39]		28.9 + 4.3 years	Embryos	11	49	22.4%

#—reference study from included studies in this review; FP—fertility preservation; PR—primary research; SR—systematic review.

**Table 2:** The usage of cryopreserved semen by men after the completion of cancer treatment.

Author/Year (Type of Study)	Participant Age Range (Years) or Mean/Median	Number of Used Materials/Return for Sperm	Number of Patients Who Cryopreserved Sperm	Return/ Usage Rate
Ferrari 2016 (SR) [45]	mean/median <35 (27 studies) median age 36 (1 study) mean age 37 (1 study) mean age 50 (1 study)	974	11,798	8.3%
Vomstein 2021 (PR) [41]	mean age 28.7	29	545 *	5.3%
Yamashita 2021 (PR) [42]	35–51 at the end of treatment (median age 36)	28	133	21.1%
Sonnenburg 2015 (PR)# [47]	16–45 (median age at diagnosis 28.4)	11	61	18.0%
Sankara-Narayana 2019 (PR) [43]	12.8–67 (median age 29)	190	2717 **	7%
Depalo 2016 (PR)# [44]	mean age 29.23 ± 7.95	19	721	2.6%
Muller 2016 (PR)# [46]	mean age 29 ± 8	96	898	10.7%
Johnson 2013 (PR)# [50]	mean age 9.7 ± 10.5	36	423	8.5%
Van der Kaaij 2014 (PR) [48]	15–69 (median age 31)	78	363	21.5%
Žáková 2014 (PR) [49]	13–64 (median age 28)	34	523	6.5%

* 545 men including 254 with testicular cancer, 156 with haematological cancer, 42 with solid tumours, 29 with sarcomas, and 64 patients with benign lesions. ** 2717 men including 2085 cancer patients, 234 with non-cancerous disease, and 398 healthy men from the control group. #—reference study from included studies in this review; PR—primary research; SR—systematic review.

**Table 3:** Disposal of unused reproductive material as part of fertility preservation in cancer patients.

Author/Year (Type of Study)	Number of Patients Who Cryopreserved Material	Reproductive Material	Disposal of Unused Cryopreserved Material		Usage Rate
			Decision	Result	
Ferrari 2016 (SR) [45]	4291	Sperm	Discard	16%	8.3%
Sankara-Narayana 2019 (PR) [43]	2717 *	Sperm	Discard due to retained/recovered fertility or sperm production	35.9%	7%
			Lost to follow-up	30.8%	
			Discard due to death	26.3%	
Moravek 2018 (PR) [36]	204	Oocytes and embryos	Ongoing storage	34.3%	10.3%
			Donate to research	33.8%	
			Donate to another couple or family member	24.5%	
			Discard	7.4%	
Muller 2016 (PR) # [46]	898	Sperm	Ongoing storage	55.4%	10.7%
			Discard upon request (death, retained/recovered fertility, spontaneous pregnancy, no desire to have children)	33.9%	
Johnson 2013 (PR) # [50]	423	Sperm	Ongoing storage	42.8%	8.5%
			Electively discarded	30.5%	
			Failed to bank a sample	10.6%	
			Discard due to death	7.6%	

* 2717 men including 2085 cancer patients, 234 non-cancer patients, and 398 healthy men in the control group. #—reference study from included studies in this review; PR—primary research; SR—systematic review.

## Data Availability

All data are available from the corresponding author.

## Supplementary Materials

The following supporting information can be downloaded at: https://www.mdpi.com/article/10.3390/cancers15225348/s1, Table S1. List of studies included and excluded after full-text analysis.

## Abbreviations

ART	assisted reproductive technology
FP	fertility preservation
GnRHa	gonadotropin-releasing hormone agonist
ICD-11: GA31	International Classification of Diseases 11th Revision, female infertility
OTT	ovarian tissue transplantation
PR	primary research
SR	systematic review
