# Supplementary material for: The Usage of Cryopreserved Reproductive Material in Cancer Patients Undergoing Fertility Preservation Procedures
Source: Cancers (Basel). 2023 Nov 9;15(22):5348. doi: 10.3390/cancers15225348 (PMC10670543; doi:10.3390/cancers15225348)
Supplement: Supplementary file 1 [file cancers-15-05348-s001.zip › cancers-2692894-supplementary.pdf]

Search strategy Medline (via PubMed)

ID	Keyword	Result
#51	Search: #43 AND #48 Filters: in the last 10 years	588
#50	Search: #43 AND #48	824
#49	Search: ((((((((((study* OR trial* OR trail* OR experiment*[Title/Abstract]))) AND ((control OR random* OR blind* OR mask*[Title/Abstract])))) OR (("Randomized Controlled Trial" [Publication Type] OR "Randomized Controlled Trials as Topic"[Mesh] OR "Controlled Clinical Trial")))))))	4,134,604
#48	Search: #43 AND #46 Filters: in the last 10 years	164
#47	Search: #43 AND #46	178
#46	Search: #44 OR #45	384,641
#45	Search: ((((((((metaanalysis[Title/Abstract]) OR Meta-Analysis[Title/Abstract]) OR "Meta-Analysis" [Publication Type]))))))	265,798
#44	Search: (((((((systematic[Title]) AND ((Review[Title/Abstract]) OR "Review" [Publication Type])))))))	232,122
#43	Search: #6 AND #42	5,884
#42	Search: #15 OR #22 OR #27 OR #33 OR #37 OR #41	23,520
#41	Search: #38 OR #39 OR #40	1,875
#40	Search: ovarian tissue freez*[Title/Abstract]	60
#39	Search: ovarian tissue cryopreserv*[Title/Abstract]	876
#38	Search: ovarian tissue cryopreservation[MeSH Terms]	1,624
#37	Search: #34 OR #35 OR #36	469
#36	Search: testicular tissue freez*[Title/Abstract]	12
#35	Search: testicular tissue cryopreserv*[Title/Abstract]	113
#34	Search: Testicular tissue cryopreservation[MeSH Terms]	430
#33	Search: #28 OR #29 OR #30 OR #31 OR #32	7,891
#32	Search: semen freez*[Title/Abstract]	212
#31	Search: semen cryopreserv*[Title/Abstract]	714
#30	Search: sperm freez*[Title/Abstract]	392
#29	Search: sperm cryopreserv*[Title/Abstract]	1,432
#28	Search: sperm cryopreservation[MeSH Terms]	7,176
#27	Search: #23 OR #24 OR #25 OR #26	9,210
#26	Search: embryo vitrificat*[Title/Abstract]	165
#25	Search: embryo freez*[Title/Abstract]	338
#24	Search: embryo cryopreserv*[Title/Abstract]	957
#23	Search: embryo cryopreservation[MeSH Terms]	8,699
#22	Search: #16 OR #17 OR #18 OR #19 OR #20 OR #21	5,126
#21	Search: fertilized oocytes cryopreserv*[Title/Abstract]	20
#20	Search: oocyte vitrificat*[Title/Abstract]	451
#19	Search: oocyte cryoconserv*[Title/Abstract]	31
#18	Search: oocyte freez*[Title/Abstract]	140
#17	Search: oocyte cryopreserv*[Title/Abstract]	1,009
#16	Search: oocyte cryopreservation[MeSH Terms]	4,687
#15	Search: #7 OR #8 OR #9 OR #10 OR #11 OR #12 OR #13 OR #14	8,464
#14	Search: fertility manag*[Title/Abstract]	299

#13	Search: oncofertility[Title/Abstract]	725
#12	Search: sterility prevent*[Title/Abstract]	11
#11	Search: infertility prevent*[Title/Abstract]	65
#10	Search: fertility secur*[Title/Abstract]	1,204
#9	Search: fertility protect*[Title/Abstract]	97
#8	Search: fertility preserv*[Title/Abstract]	5,442
#7	Search: fertility preservation[MeSH Terms]	3,794
#6	Search: #1 OR #2 OR #3 OR #4 OR #5	4,933,281
#5	Search: tumor*[Title/Abstract]	1,749,481
#4	Search: malignan*[Title/Abstract]	684,627
#3	Search: neoplasm*[Title/Abstract]	311,339
#2	Search: cancer*[Title/Abstract]	2,245,500
#1	Search: cancer[MeSH Terms]	3,837,821

Search strategy Cochrane Library

ID	Keyword	Result
#1	MeSH descriptor: [Neoplasms] explode all trees	111,265
#2	(cancer*):ti,ab,kw	197,315
#3	(neoplasm*):ti,ab,kw	106,339
#4	(malignan*):ti,ab,kw	32,006
#5	(tumor*):ti,ab,kw	80,084
#6	#1 OR #2 OR #3 OR #4 OR #5	264,254
#7	MeSH descriptor: [Fertility Preservation] explode all trees	67
#8	(fertility preserv*):ti,ab,kw	429
#9	(fertility protect*):ti,ab,kw	134
#10	(fertility secur*):ti,ab,kw	82
#11	(infertility prevent*):ti,ab,kw	807
#12	(sterility prevent*):ti,ab,kw	62
#13	(Oncofertility):ti,ab,kw	18
#14	(fertility manag*):ti,ab,kw	506
#15	#7 OR #8 OR #9 OR #10 OR #11 OR #12 OR #13 OR #14	1,828
#16	(oocyte cryopreserv*):ti,ab,kw	484
#17	(oocyte freez*):ti,ab,kw	279
#18	(oocyte cryoconserv*):ti,ab,kw	2
#19	(oocyte vitrificat*):ti,ab,kw	237
#20	(fertilized oocytes cryopreserv*):ti,ab,kw	84
#21	#16 OR #17 OR #18 OR #19 OR #20	750
#22	(embryo cryopreserv*):ti,ab,kw	861
#23	(embryo freez*):ti,ab,kw	572
#24	(embryo vitrificat*):ti,ab,kw	349
#25	#22 OR #23 OR #24	1,339
#26	(sperm cryopreserv*):ti,ab,kw	441
#27	(sperm freez*):ti,ab,kw	276

#28	(semen cryopreserv*):ti,ab,kw	116
#29	(semen freez*):ti,ab,kw	74
#30	#26 OR #27 OR #28 OR #29	632
#31	(testicular tissue cryopreserv*):ti,ab,kw	6
#32	(testicular tissue freez*):ti,ab,kw	5
#33	#31 OR #32	7
#34	(ovarian tissue cryopreserv*):ti,ab,kw	79
#35	(ovarian tissue freez*):ti,ab,kw	47
#36	#34 OR #35	104
#37	#15 OR #21 OR #25 OR #30 OR #33 OR #36	3,341
#38	#6 AND #37	547
#39	#6 AND #37 with Cochrane Library publication date from Jun 2013 to Jun 2023, in Cochrane Reviews	35
#40	#6 AND #37 with Publication Year from 2013 to 2023, in Trials	349

Search strategy Embase (via Ovid)

ID	Keyword	Result
1	exp malignant neoplasm/	3,656,681
2	"cancer*".ab,kw,ti.	3,075,039
3	"neoplasm*".ab,kw,ti.	217,379
4	"Malignan*".ab,kw,ti.	908,817
5	"Tumor*".ab,kw,ti.	2,233,019
6	1 or 2 or 3 or 4 or 5	5,545,534
7	exp fertility preservation/	7,706
8	"fertility preserv*".ab,kw,ti.	10,039
9	"fertility protect*".ab,kw,ti.	194
10	"fertility secur*".ab,kw,ti.	2
11	"infertility prevent*".ab,kw,ti.	94
12	"sterility prevent*".ab,kw,ti.	4
13	Oncofertility.ab,kw,ti.	1,133
14	"fertility manag*".ab,kw,ti.	323
15	7 or 8 or 9 or 10 or 11 or 12 or 13 or 14	12,517
16	exp oocyte cryopreservation/	982
17	"oocyte cryopreserv*".ab,kw,ti.	1,985
18	"oocyte freez*".ab,kw,ti.	317
19	"oocyte cryoconserv*".ab,kw,ti.	1
20	"oocyte vitrificat*".ab,kw,ti.	946
21	"fertilized oocytes cryopreserv*".ab,kw,ti.	4
22	17 or 18 or 19 or 20 or 21	2,899
23	"embryo cryopreserv*".ab,kw,ti.	1,735
24	"embryo freez*".ab,kw,ti.	646
25	"embryo vitrificat*".ab,kw,ti.	395
26	23 or 24 or 25	2,613

27	exp sperm count/	2,927
28	"sperm cryopreserv*".ab,kw,ti.	2,102
29	"sperm freez*".ab,kw,ti.	601
30	"Semen cryopreserv*".ab,kw,ti.	1,020
31	"semen freez*".ab,kw,ti.	279
32	27 or 28 or 29 or 30 or 31	6,348
33	exp testis tissue/	4,186
34	"testicular tissue cryopreserv*".ab,kw,ti.	177
35	"Testicular tissue freez*".ab,kw,ti.	32
36	33 or 34 or 35	4,284
37	exp ovary tissue/	4,365
38	"ovarian tissue cryopreserv*".ab,kw,ti.	1,487
39	"ovarian tissue freez*".ab,kw,ti.	105
40	37 or 38 or 39	5,206
41	15 or 22 or 26 or 32 or 36 or 40	28,344
42	6 and 41	10,753
43	limit 42 to ((consensus development or meta analysis or "systematic review") and last 10 years)	376
44	limit 42 to ((clinical trial or randomized controlled trial or controlled clinical trial or multicenter study) and last 10 years)	325

Table 1. List of studies included and excluded after full-text analysis

No.	Authors, Title, Journal	Full text status	Reasons
**SECONDARY STUDIES**			
**1**	**Bonardi B., Massarotti C., Bruzzone M. et al. (2020). Efficacy and safety of controlled ovarian stimulation with or without letrozole co-administration for fertility preservation: a systematic review and meta-analysis. Front. Oncol. 10: 574669**	**INCLUDED**	**–**
**2**	**Ferrari S., Paffoni A., Filippi F. et al. (2016). Sperm cryopreservation and reproductive outcome in male cancer patients: a systematic review. Reprod. Biomed. Online. 33(1): 29-38**	**INCLUDED**	**–**
**3**	**Fraison E., Huberlant S., Labrune E. et al. (2023). Live birth rate after female fertility preservation for cancer or haematopoietic stem cell transplantation: a systematic review and meta-analysis of the three main techniques; embryo, oocyte and ovarian tissue cryopreservation. Hum. Reprod. 38(3): 489-502.**	**INCLUDED**	**–**
**4**	**Ozcan M. C., Snegovskikh V., Adamson G. D. (2022). Oocyte and embryo cryopreservation before gonadotoxic treatments: Principles of safe ovarian stimulation, a systematic review. Women's Health (Lond). 18: 17455065221074886**	**INCLUDED**	**–**
**5**	**Sheshpari S., Shahnazi M., Mobarak H. et al. (2019). Ovarian function and reproductive outcome after ovarian tissue transplantation: a systematic review. J. Transl. Med. 17(1): 1-15**	**INCLUDED**	**–**
**6**	**Wang S. S., Loong H., Chung J. P. et al. (2020). Preservation of fertility in premenopausal patients with breast cancer. Hong Kong Med. J. 26(3): 216-226**	**INCLUDED**	**–**

7	**Xu Z., Ibrahim S., Burdett S. et al. (2023). Long term pregnancy outcomes of women with cancer following fertility preservation: A systematic review and meta-analysis. Eur. J. Obstet. Gynecol. Reprod. Biol. 281: 41-48**	**INCLUDED**	–
8	Alexander V. M., Martin C. E., Schelble A. P. et al. (2021). Ovarian stimulation for fertility preservation in women with cancer: A systematic review and meta-analysis comparing random and conventional starts. J. Gynecol. Obstet. Hum. Reprod. 50(8): 102080	EXCLUDED	I
9	Amzai G., Stojanovic A. (2013). Preservation of fertility and of reproduction ability in lymphoma patients. Maced. J. Med. Sci. 6(2):194-201	EXCLUDED	M
10	Anbari F., Khalili M. A., Mahaldashtian M. et al. (2022). Fertility preservation strategies for cancerous women: An updated review. Turk. J. Obstet. Gynecol. 19(2): 152-161	EXCLUDED	M
11	Arcieri M., Cianci S., Martinelli C. et al. (2022). Uterine Smooth Muscle Tumor of Uncertain Malignant Potential (STUMP) Treated with Conservative Surgery: Systematic Review of Reproductive Outcomes. Clin. Exp. Obstet. Gynecol. 49(12), 267	EXCLUDED	I, M
12	Arecco L., Blondeaux E., Bruzzone M. et al. (2022). Safety of fertility preservation techniques before and after anticancer treatments in young women with breast cancer: a systematic review and meta-analysis. Hum. Reprod. 37(5): 954-968	EXCLUDED	I
13	Bastings L., Beerendonk C. C. M., Westphal J. R. et al. (2013). Autotransplantation of cryopreserved ovarian tissue in cancer survivors and the risk of reintroducing malignancy: a systematic review. Hum. Reprod. Update. 19(5), 483-506	EXCLUDED	M
14	Chen C. N., Chang L. T., Chen C. H. et al. (2022). Fertility preservation for women with breast cancer before chemotherapy: a systematic review and meta-analysis. Reprod. Biomed. Online. 44(2): 357-369	EXCLUDED	I, M
15	Clasen N. H., van der Perk M. M., Neggers S. J. et al. (2023). Experiences of Female Childhood Cancer Patients and Survivors Regarding Information and Counselling on Gonadotoxicity Risk and Fertility Preservation at Diagnosis: A Systematic Review. Cancers. 15(7): 1946	EXCLUDED	P, I
16	Corkum K. S., Rhee D. S., Wafford Q. E. et al. (2019). Fertility and hormone preservation and restoration for female children and adolescents receiving gonadotoxic cancer treatments: a systematic review. J. Pediatr. Surg. 54(11): 2200-2209	EXCLUDED	I
17	Dhonnabháin B.N., Elfaki N., Fraser K. et al. (2022). A comparison of fertility preservation outcomes in patients who froze oocytes, embryos, or ovarian tissue for medically indicated circumstances: a systematic review and meta-analysis. Fertil. Steril. 117(6): 1266-1276	EXCLUDED	P, I
18	Diaskosavvas M., Fasoulakis Z., Ntounis T. et al. (2021). A Potential Pathogenic Link Between Cancer of Female Reproductive System and Infertile Women Treated With Assisted Reproduction Techniques. In Vivo. 35(3): 1393-1399	EXCLUDED	P, I
19	Dieamant F., Petersen C.G., Vagnini L.D. et al. (2021). Impact of Intracytoplasmic Morphologically Selected Sperm Injection (IMSI) on Birth Defects: A Systematic Review and Meta-Analysis. JBRA Assisted Reprod. 25(3):466-472	EXCLUDED	I
20	Eijkenboom L., Saedt E., Ziestse C. et al. (2022). Strategies to safely use cryopreserved ovarian tissue to restore fertility after cancer: a systematic review. Reprod. Biomed. Online. 5(4):763-778	EXCLUDED	P, I
21	Elfaki N., Lutaaya N., Hardiman P.J. et al. (2021). Oocyte cryopreservation for social reasons versus fertility preservation; a systematic review. BJOG. 128: 238	EXCLUDED	Lack of access

22	Faizal A.M., Sugishita Y., Suzuki-Takahashi Y. et al. (2022). Twenty-first century oocyte cryopreservation—in vitro maturation of immature oocytes from ovarian tissue cryopreservation in cancer patients: A systematic review. Womens Health (London)	EXCLUDED	M
23	Feng Y., Zhang Z., Lou T. et al. (2018). The safety of fertility preservation for microinvasive cervical adenocarcinoma: a meta-analysis and trial sequential analysis. Arch. Gynecol. Obstet. 298(3): 465-475	EXCLUDED	I, M
24	Fernandez-Montoli M.E., Sabadell J., Martinez-Garcia J.M. et al. (2018). Fertility-sparing treatment for atypical endometrial hyperplasia and endometrial cancer. Adv. Ther. 38(5): 2717-2731	EXCLUDED	Lack of access
25	Gilbert K., Nangia A. K., Dupree J. M. et al. (2018). Fertility preservation for men with testicular cancer: is sperm cryopreservation cost effective in the era of assisted reproductive technology? Urol. Oncol. 36(3): 92.e1-92.e9	EXCLUDED	I, M
26	Gilbert K., Nangia A., Mehta, A. (2017). Cost-effectiveness of fertility preservation in testis cancer patients. J. Urol. 197(4S): 275	EXCLUDED	Lack of access
27	Heiser P., Wei S., Dutia R. et al. (2016). Characteristics of successful fertility preservation programs for cancer patients. J. Clin. Oncol. 34(15)	EXCLUDED	Lack of access
28	Khattak H., Malhas R., Craciunas L. et al. (2022). Fresh and cryopreserved ovarian tissue transplantation for preserving reproductive and endocrine function: a systematic review and individual patient data meta-analysis. Hum. Reprod. Update. 28(3): 400-416	EXCLUDED	I, M
29	Khattak H., Malhas R., Craciunas L. et all. (2021). Reproductive and endocrine outcomes after fresh and frozen-thawed ovarian tissue transplantation based on age and anti-cancer therapy: A systematic review and individual patient data meta-analysis. Hum. Reprod. 36: i98-i99	EXCLUDED	Lack of access
30	Khattak H., Woodman H., Gallos I. et al. (2022). Experiences of females undergoing ovarian cryopreservation: A systematic review and thematic synthesis. An Int. J. Obstetric. Gynaecol. 129(0): 182-183	EXCLUDED	Lack of access
31	Khattak H., Woodman H., Gallos I. et al. (2022). Experiences of young girls and women undergoing ovarian tissue cryopreservation: a systematic review and thematic synthesis. J. Psychosom. Obstet. Gynaecol. 43(4): 502-516	EXCLUDED	Lack of access
32	Kim H., Kim S. K., Lee J. R. et al. (2017). Fertility preservation for patients with breast cancer: The Korean Society for Fertility Preservation clinical guidelines. Clin. Exp. Reprod. Med. 44(4): 181-186	EXCLUDED	M
33	Logan S., Perz J., Ussher J. et al. (2018). Clinician provision of oncofertility support in cancer patients of a reproductive age: a systematic review. Psychooncology. 27(3): 748-756	EXCLUDED	I, M
34	Logan S., Perz J., Ussher J. M. et al. (2019). Systematic review of fertility-related psychological distress in cancer patients: Informing on an improved model of care. Psychooncology. 28(1): 22-30	EXCLUDED	I, M
35	Long C. J., Ginsberg J. P., Kolon T. F. (2016). Fertility preservation in children and adolescents with cancer. Urology. 91: 190-196	EXCLUDED	M
36	Lucchini S. M., Esteban A., Nigra M. A. et al. (2021). Updates on conservative management of endometrial cancer in patients younger than 45 years. Gynecol. Oncol. 161(3): 802-809	EXCLUDED	I
37	Lutaaya N., Dhonnabhain N., Elfaki N. et al. (2022). A systematic review and meta-analysis of ovarian stimulation outcomes in cancer patients opting for oocyte cryopreservation prior to treatment compared to social egg-freezers. Hum. Reprod. 37: 107.437	EXCLUDED	Lack of access

38	Ogouma L., Berthaut I., Lévy R. et al. (2022). Testicular sperm extraction (TESE) outcomes in the context of malignant disease: a systematic review. Asian J. Androl. 24(6): 584-590	EXCLUDED	M
39	Pontre J.C. (2020). A systematic review of fertility outcomes after uterine preserving management for endometrial cancer and hyperplasia. Gynecol. Oncology. 159: 199-200	EXCLUDED	Lack of access
40	Qin N., Wan Z., Kang Y. et al. (2023). Effectiveness and feasibility of online fertility preservation decision aids for young female patients with cancer: a systematic review protocol. BMJ Open. 13(3), e070294	EXCLUDED	I, M
41	Rosendahl M., Greve T., Andersen C. Y. (2013). The safety of transplanting cryopreserved ovarian tissue in cancer patients: a review of the literature. J. Assist. Reprod. Genet. 30(1): 11-24	EXCLUDED	I, M
42	Salama M., Woodruff T. K. (2015). New advances in ovarian autotransplantation to restore fertility in cancer patients. Cancer Metastasis Rev. 34(4): 807-822	EXCLUDED	M
43	Santos M. L., Pais A. S., Almeida Santos T. (2021). Fertility preservation in ovarian cancer patients. Gynecol. Endocrin. 37(6): 483-489	EXCLUDED	M
44	Senra J. C., Roque M., Talim M. C. et al. (2018). Gonadotropin-releasing hormone agonists for ovarian protection during cancer chemotherapy: systematic review and meta-analysis. Ultrasound Obstet. Gynecol. 51(1): 77-86	EXCLUDED	I
45	Silvestris E., Paradiso A. V., Minoia C. et al. (2022). Fertility preservation techniques in cervical carcinoma. Medicine (Baltimore). 101(17): e29163-e29163	EXCLUDED	I, M
46	Speller B., Micic S., Daly C. et al. (2019). Oncofertility decision support resources for women of reproductive age: systematic review. JMIR Cancer. 5(1): e12593	EXCLUDED	I, M
47	Strowitzki T., Bruckner T., Roesner S. (2021). Maternal and neonatal outcome and children's development after medically assisted reproduction with in-vitro matured oocytes—A systematic review and meta-analysis. Hum. Reprod. Update. 27(3): 460-473	EXCLUDED	P, I
48	Sukur Y.E., Ates C., Sacinti K.G. et al. (2022). Random-start versus conventional ovarian hyperstimulation for fertility preservation in female cancer patients: a systematic review and meta-analysis. Fretil. Steril. 4: e238-e239	EXCLUDED	Lack of access
49	Taylor J. F., Ott M. A. (2016). Fertility preservation after a cancer diagnosis: a systematic review of Adolescents', Parents', and providers' perspectives, experiences, and preferences. J. Pediatr. Adolesc. Gynecol. 29(6): 585-598	EXCLUDED	M
50	Tomao F., Di Pinto A., Sassu C. M. et al. (2018). Fertility preservation in ovarian tumours. Ecancermedicalscience. 12	EXCLUDED	I, M
51	Valipour A., Osowski S., Rey J. et al. (2019). Semen cryopreservation in adolescent and adult men undergoing fertility compromising cancer treatment: A systematic review. Andrologia. 51(11): e13392	EXCLUDED	I, M
52	Viviani S., Caccavari V., Gerardi C. et al. (2021). Male and Female Fertility: Prevention and Monitoring Hodgkin'Lymphoma and Diffuse Large B-Cell Lymphoma Adult Survivors. A Systematic Review by the Fondazione Italiana Linfomi. Cancers (Basel). 13(12): 2881	EXCLUDED	I, M
53	Wang Y., Anazodo A., Logan S. (2019). Systematic review of fertility preservation patient decision aids for cancer patients. Psychooncology, 28(3): 459-467	EXCLUDED	I
54	White R., Odendaal J., Wilson A. et al. (2022). Fertility preservation, its effectiveness and safety in pre-menopausal women with breast cancer. Inter. J. Obscetrics Gynecol. 129(0): 181	EXCLUDED	Lack of access

55	Zdenkowski N., Butow P., Tesson S. et al. (2016). A systematic review of decision aids for patients making a decision about treatment for early breast cancer. Breast. 26: 31-45	EXCLUDED	I
56	Zhang H. F., Jiang Q. H., Huang G. Y. et al. (2021). The educational program for healthcare providers regarding fertility preservation for cancer patients: a systematic review. J. Cancer. Educ. 36(3):452-462	EXCLUDED	I
**PRIMARY STUDIES**			
**1**	**Courbiere B., Decanter C., Bringer-Deutsch S. et al. (2013). Emergency IVF for embryo freezing to preserve female fertility: a French multicentre cohort study. Hum. Reprod. 28(9): 2381-2388**	**INCLUDED**	**–**
**2**	**Marklund A., Lundberg F. E., Eloranta S. et al. (2021). Reproductive outcomes after breast cancer in women with vs without fertility preservation. JAMA Oncol. 7(1), 86-91**	**INCLUDED**	**–**
**3**	**Moravek M. B., Confino R., Smith K. N. et al. (2018). Long-term outcomes in cancer patients who did or did not pursue fertility preservation. Fertil. Steril. 109(2): 349-355**	**INCLUDED**	**–**
**4**	**Porcu E., Cipriani L., Dirodi M. et al. (2022). Successful pregnancies, births, and children development following oocyte cryostorage in female cancer patients during 25 years of fertility preservation. Cancers (Basel). 14(6): 1429**	**INCLUDED**	**–**
**5**	**Rodriguez-Wallberg K. A., Marklund A., Lundberg F. et al. (2019). A prospective study of women and girls undergoing fertility preservation due to oncologic and non-oncologic indications in Sweden–Trends in patients' choices and benefit of the chosen methods after long-term follow up. Acta Obstet. Gynecol. Scand. 98(5): 604-615**	**INCLUDED**	**–**
**6**	**Shankara-Narayana N., Di Pierro I., Fennell C. et al. (2019). Sperm cryopreservation prior to gonadotoxic treatment: experience of a single academic centre over 4 decades. Hum. Reprod. 34(5): 795-803**	**INCLUDED**	**–**
**7**	**Van der Kaaij M. A. E., van Echten-Arends J., Heutte N. et al. (2014). Cryopreservation, semen use and the likelihood of fatherhood in male Hodgkin lymphoma survivors: an EORTC-GELA Lymphoma Group cohort study. Hum. Reprod. 29(3): 525-533**	**INCLUDED**	**–**
**8**	**Vomstein K., Reiser E., Pinggera G. M. et al. (2021). Sperm banking before gonadotoxic treatment: is it worth the effort?. Asian J. Androl. 23(5): 490-494**	**INCLUDED**	**–**
**9**	**Yamashita S., Kakimoto K., Uemura M. et al. (2021). Fertility and reproductive technology use in testicular cancer survivors in Japan: A multi-institutional, cross-sectional study. Int. J. Urol. 28(10): 1047-1052**	**INCLUDED**	**–**
**10**	**Žáková J., Lousová E., Ventruba P. et al. (2014). Sperm cryopreservation before testicular cancer treatment and its subsequent utilization for the treatment of infertility. Scientific World Journal, 2014: 575978**	**INCLUDED**	**–**
11	Benoit A., Grynberg M., Morello R. et al. (2020). Does a web-based decision aid improve informed choice for fertility preservation in women with breast cancer (DECISIF)? Study protocol for a randomised controlled trial. BMJ Open. 10(2): e031739	EXCLUDED	I
**12**	Borgmann-Staudt A., Kunstreich M., Schilling R. et al. (2019). Fertility knowledge and associated empowerment following an educational intervention for adolescent cancer patients. Psychooncology. 28(11): 2218-2225	EXCLUDED	I

13	Cohen Y., Tannus S., Volodarsky-Perel. et al. (2020). Added benefit of immature oocyte maturation for fertility preservation in women with malignancy. Reprod. Sci. 27: 2257-2264	EXCLUDED	I
14	Condorelli M., Bruzzone M., Ceppi M. et al. (2021). Safety of assisted reproductive techniques in young women harboring germline pathogenic variants in BRCA1/2 with a pregnancy after prior history of breast cancer. ESMO Open. 6(6): 100300	EXCLUDED	I, M
15	Dolmans M. M., Marotta M. L., Pirard C. et al. (2014). Ovarian tissue cryopreservation followed by controlled ovarian stimulation and pick-up of mature oocytes does not impair the number or quality of retrieved oocytes. J. Ovarian. Res. 7: 80	EXCLUDED	I, M
16	Ehrbar V., Germeyer A., Nawroth F. et al. (2021). Long-term effectiveness of an online decision aid for female cancer patients regarding fertility preservation: Knowledge, attitude, and decisional regret. Acta Obstet. Gynecol. Scand. 100(6): 1132-1139	EXCLUDED	I
17	Ehrbar V., Urech C., Rochlitz C. et al. (2019). Randomized controlled trial on the effect of an online decision aid for young female cancer patients regarding fertility preservation. Hum. Reprod. 34(9): 1726-1734	EXCLUDED	I
18	Garvelink M. M., Ter Kuile M. M., Louwé L. A. et al. (2017). Feasibility and effects of a decision aid about fertility preservation. Hum. Fertil. (Camb). 20(2): 104-112	EXCLUDED	I
19	Gat I., Toren A., Hourvitz A. et al. (2014). Sperm preservation by electroejaculation in adolescent cancer patients. Pediatr. Blood Cancer. 61(2): 286-290	EXCLUDED	I, M
20	Hulsbosch S., Koskas M., Tomassetti C. et al. (2018). A real-life analysis of reproductive outcome after fertility preservation in female cancer patients. Gynecol. Obstet. Invest. 83(2): 156-163	EXCLUDED	M
21	Imbert R., Moffa F., Tsepelidis S. et al. (2014). Safety and usefulness of cryopreservation of ovarian tissue to preserve fertility: a 12-year retrospective analysis. Hum. Reprod. 29(9): 1931-1940	EXCLUDED	M
22	Jensen A. K., Macklon K. T., Fedder J. et al. (2017). 86 successful births and 9 ongoing pregnancies worldwide in women transplanted with frozen-thawed ovarian tissue: focus on birth and perinatal outcome in 40 of these children. J. Assist. Reprod. Genet. 34: 325-336	EXCLUDED	M
23	Kawwass J. F., Shandley L. M., Boulet S. L. et al. (2020). Oncologic oocyte cryopreservation: national comparison of fertility preservation between women with and without cancer. J. Assist. Reprod. Genet. 37(4): 883-890	EXCLUDED	M
24	Kim S., Lee Y., Lee S. et al. (2018). Ovarian tissue cryopreservation and transplantation in patients with cancer. Obstet. Gynecol. Sci. 61(4): 431-442	EXCLUDED	M
25	Lantsberg D., Farhi A., Zaslavsky-Paltiel I. et al. (2019). Deliveries following fertility preservation by ovarian tissue cryopreservation without autotransplantation—what should be expected J. Assist. Reprod. Genet. 36(2): 335-340	EXCLUDED	I, M
26	Li Y., Cai X., Dong B. et al. (2022). The Impact of Malignancy on Assisted Reproductive Outcomes for Cancer Survivors: A Retrospective Case–Control Study. Front. Oncol. 12: 941797	EXCLUDED	M
27	Li Y., Zhang J., Zhang H. et al. (2020). Importance and safety of autologous sperm cryopreservation for fertility preservation in young male patients with cancer. Medicine (Baltimore), 99(15): e19589	EXCLUDED	I
28	Luke B., Brown M. B., Spector L. G. et al. (2016). Embryo banking among women diagnosed with cancer: a pilot population-based study in New York, Texas, and Illinois. J. Assist. Reprod. Genet. 33(5): 667-674	EXCLUDED	M

29	Øvlisen A. K., Jakobsen L. H., Eloranta S. et al. (2021). Parenthood rates and use of assisted reproductive techniques in younger Hodgkin lymphoma survivors: A Danish population-based study. J. Clin. Oncol. 39(31): 3463-3472	EXCLUDED	M
30	Rose B. I., Nguyen K. (2023). The effect of in vitro maturation (IVM) protocol changes on measures of oocyte/embryo competence. Reprod. Med. 4(1): 65-73	EXCLUDED	I, M
31	Rotker K., Vigneswaran H., Omil-Lima D. et al. (2017). Efficacy of standardized nursing fertility counseling on sperm banking rates in cancer patients. Urology. 104: 90-96	EXCLUDED	M
32	Sommerhäuser G., Borgmann-Staudt A., Astrahantseff K. et al. (2021). Health outcomes in offspring born to survivors of childhood cancers following assisted reproductive technologies. J. Cancer Surviv. 15(2): 259-272	EXCLUDED	I, M
33	Ussher J. M., Perz J., Hawkey A. J. (2021). A randomized controlled evaluation of an educational resource to address fertility concerns after cancer. PsychoOncology. 30(9): 1442-1448	EXCLUDED	I, M
34	Van der Ven H., Liebenthron J., Beckmann M. et al. (2016). Ninety-five orthotopic transplantations in 74 women of ovarian tissue after cytotoxic treatment in a fertility preservation network: tissue activity, pregnancy and delivery rates. Hum. Reprod. 31(9): 2031-2041	EXCLUDED	M
35	Virant-Klun I., Bedenk J., Jancar N. (2021). In vitro maturation of immature oocytes for fertility preservation in cancer patients compared to control patients with fertility problems in an in vitro fertilization program. Radiol. Oncol. 56(1): 119-128	EXCLUDED	I, M
36	Virant-Klun I., Bedenk J., Jancar N. (2022). Maturation of immature oocytes for fertility preservation in cancer patients compared to control patients with fertility problems in an fertilization program. Radiol. Oncol. 56(1): 119-128	EXCLUDED	I, M
37	Vuković P., Kasum M., Raguž J. et al. (2019). Fertility preservation in young women with early-stage breast cancer. Acta Clin. Croat. 58(1): 147-156	EXCLUDED	M

P – population; I – intervention; M – methodology

Methodology of included studies

Secondary studies (n=7) and references (n=4)

- Fraison 2023 – a systematic review with a meta-analysis of 73 observational studies, which estimated the live birth rate in the case of oocyte, embryo and ovarian tissue cryopreservation among women undergoing cancer treatment;
- Xu 2023 – a systematic review with a meta-analysis of 26 observational studies evaluating long-term reproductive outcomes when fertility preservation (FP) measures are implemented in women diagnosed with cancer;
- Ozcan 2022 – a systematic review of 2 RCTs and 60 cohort studies and 20 additional expert opinions that established the safety of fertility preservation by ovarian stimulation as part of cryopreservation of oocytes or embryos:
    - Dolmas 2015 (reference) – a retrospective observational study of cancer patients undergoing ovarian stimulation, from whom embryos were collected and cryopreserved in order to preserve fertility in 1997-2014;
- Bonardi 2020 – a systematic review with a meta-analysis of 11 cohort studies comparing the efficacy and safety of controlled ovarian stimulation (COS) with or without concomitant administration of letrozole in patients with breast cancer;

  - Johnson 2013a (reference) – a cohort study evaluating the efficacy of controlled ovarian stimulation for cryopreservation of oocytes/embryos in cancer patients in 2005-2012;
- Wang 2020 – a systematic review, summarizing methods of preserving fertility in patients with breast cancer:
  - Cobo 2018 (reference) – a multicenter, retrospective cohort study determining the efficacy of using vitrified oocytes for fertilization of women undergoing cancer treatment in 2007-2018;
  - Martinez 2014 (reference) – a prospective cohort study presenting the results of cancer patients undergoing oocyte vitrification for fertility preservation in 2007-2012;
- Sheshpari 2019 – a systematic review of 25 cohort studies and case reports that summarized reproductive outcomes for ovarian tissue harvesting, cryopreservation and transplantation in cancer-treated women;
- Ferrari 2016 – a systematic review with a meta-analysis of 30 observational studies, presenting data on the use and efficacy of programs aimed at semen preservation among men with cancer;

Primary studies (n=10) and references (n=5)

- Porcu 2022 – a prospective cohort study evaluating the usefulness of cryopreserved oocytes as part of fertility preservation in oncological patients compared to the treatment of infertility of people without diagnosed cancer in the perspective of 25 years (1996-2021);
- Marklund 2021 – a cohort study with a control group, evaluating long-term reproductive outcomes in the case of implementation or lack of implementation of fertility preservation measures in women diagnosed with breast cancer:
  - Marklund 2020 (reference) – a multicenter, prospective cohort study assessing the efficacy and safety of controlled ovarian stimulation for fertility preservation in Swedish women with breast cancer in 1995-2017;
- Vomestein 2021 – a retrospective cohort study in which the results of semen quality before gonadotoxic treatment and the usage rates of cryopreserved material as part of ART in cancer patients and men with non-cancer diseases in 2008-2018 were analyzed and compared;
- Yamashita 2021 – a cross-sectional study evaluating reproductive rates in the usage of cryopreserved semen collected from men with testicular cancer for fertility preservation and ART;
  - Sonnenburg 2015 (reference) – a retrospective observational study assessing utilization rate and reproductive outcomes of cryopreserved semen collected from men with testicular germ cell tumor;
- Rodriguez-Wallberg 2019 – a prospective cohort study, determining the efficacy and usage rates of cryopreserved material as part of fertility preservation in women diagnosed with cancer in relation to women with other non-cancer diseases in 1998-2018;
- Sankara-Narayana 2019 – a retrospective observational study determining the efficacy of storing cryopreserved semen from men undergoing cancer treatment or suffering from another disease, as part of the Australian fertility preservation program in 1978-2017:
  - Depolo 2016 (reference) – a retrospective observational study assessing semen parameters, sperm viability and reproductive indices in the case of using cryopreserved semen for ART in men undergoing cancer treatment in 1999-2015;
  - Muller 2016 (reference) – a retrospective observational study determining the usage rate of cryopreserved semen collected from men with cancer in 1983-2013;
  - Johnson 2013b (reference) – a retrospective cohort study comparing the disposition and semen parameters of cryopreserved spermatozoa from cancer patients compared to men treated for infertility;

- Moravek 2018 – a retrospective cohort study comparing long-term reproductive outcomes of cancer patients using fertility preservation methods compared to cancer patients who did not choose any fertility preservation method in 2005-2016;
- Van der Kaaij 2014 – a case-control study, determining the usage rate and reproductive indices of cryopreserved semen collected from men diagnosed with cancer;
- Žáková 2014 – a single-arm study presenting the outcomes of cryopreserved semen storage for fertility preservation and subsequent usage in the treatment of infertility in men with testicular cancer in 1995-2012;
- Courbiere 2013 – a retrospective cohort study, determining the efficacy of the usage of cryopreserved embryos for emergency in vitro in women undergoing cancer treatment in 1999-2011;
